# The Effect of Social Trust on Citizens’ Health Risk Perception in the Context of a Petrochemical Industrial Complex

**DOI:** 10.3390/ijerph10010399

**Published:** 2013-01-21

**Authors:** Miguel Ángel López-Navarro, Jaume Llorens-Monzonís, Vicente Tortosa-Edo

**Affiliations:** Department of Business Administration and Marketing, Jaume I University, Av. Vicent Sos Baynat, Castelló de la Plana 12071, Spain; E-Mails: jllorens@emp.uji.es (J.L.-M.); vtortosa@emp.uji.es (V.T.-E.)

**Keywords:** petrochemical industry, citizens’ health risk perception, social trust, trust in companies, trust in public institutions

## Abstract

Perceived risk of environmental threats often translates into psychological stress with a wide range of effects on health and well-being. Petrochemical industrial complexes constitute one of the sites that can cause considerable pollution and health problems. The uncertainty around emissions results in a perception of risk for citizens residing in neighboring areas, which translates into anxiety and physiological stress. In this context, social trust is a key factor in managing the perceived risk. In the case of industrial risks, it is essential to distinguish between trust in the companies that make up the industry, and trust in public institutions. In the context of a petrochemical industrial complex located in the port of Castellón (Spain), this paper primarily discusses how trust—both in the companies located in the petrochemical complex and in the public institutions—affects citizens’ health risk perception. The research findings confirm that while the trust in companies negatively affects citizens’ health risk perception, trust in public institutions does not exert a direct and significant effect. Analysis also revealed that trust in public institutions and health risk perception are essentially linked indirectly (through trust in companies).

## 1. Introduction

In recent years, the issue of environmental quality and its effect on people’s health and well-being have gained importance in academic research. The pollution associated with particular industrial sites can have negative effects on health of individuals who live nearby residential areas [1,2,3,4,5,6,7]. Beyond the awareness of residents about the cause-effect relationship between pollution and specific diseases, the potential exposure to contaminants create uncertainty around this issue resulting in a perception of risks contributing to undermine citizens’ welfare and quality of life. As Cutchin *et al.* [8] suggest, “active industrial sites, particularly those producing petrochemical products, are associated with increased stress and self-reported illness (...) psychosocial anxiety and distress often translate into physiological stress with a wide range of effects on health and well-being”. Consequently, the risk assessment in populations exposed to these hazards is an essential element in understanding health and welfare of citizens, and that assessment has not received adequate attention in the literature [8,9]. Moreover, proper management of environmental risks is critical, especially if we consider the potential for effective mitigation of psychosocial effects through the environmental (risk assessment) policy process [10].

In the field of risk research there is now general agreement that trust in risk management situations constitutes an important factor in perception and acceptance of risks [11,12]. Specifically, citizens try to form fair judgments about the risks surrounding them, but the information is often limited or they are not prepared to understand it [13]. Under such circumstances, people have to rely on others, which is why trust plays a dominant role. The importance of social trust to the understanding of risk perception has been recognized and has gained widespread attention [14,15,16,17,18,19]. In a review paper regarding trust in the risk management field [18], it is noted that 58 (57%) of the 102 articles analyzed that studied the consequences of trust, included risk perception, making it the most frequently studied consequence. However research in trust in this context is highly fragmented and it has generated conflicting results and calls for additional examination. When literature refers to the social trust, it is common to include trust in industry and trust in public institutions within the same construct (see, for example [20,21]), although some studies [15,16,22], consider separately both types of trust, as two different variables. Industry and public institutions are organizations that are responsible for risk management and communication although citizens’ trust in each one derives from a different conception. On one hand, trust in companies located in the petrochemical complex is important because they are the first responsible for managing the risks inherent to the industrial activity which themselves developed; on the other hand, trust in public institutions can also play a decisive role in the risk perception because these institutions have a regulatory role and the task of supervising the firms’ activities and preserving the environmental quality of the surroundings. Authors like Maeda and Miyahara [22] make this distinction in its analysis regarding the determinants of trust, but they estimate separate models for trust in industry and for trust in public institutions. Trumbo and McComas [15,16] simultaneously analyzed the incidence of both types of trust on the perceived risk, but they do not establish causality between the two kinds of trust. As far as we have been able to confirm, there are no studies that simultaneously address the incidence of both types of trust on citizens’ risk perception, trying to assess in turn the causal relationship between them.

In the context of a petrochemical industrial complex located in the port of Castellón (Spain), this paper firstly analyzes how trust in companies located in the complex and trust in public institutions, considered simultaneously, determine residents’ health risk perception. Within the framework of the same model, we also analyzed the relationship between the two types of trust. We argue that beyond trust in public institutions can exert a direct effect on the citizens’ risk perception, this one can also influence indirectly risk perception through its impact on trust in the companies located in the complex. This is an important issue because understanding the direct and indirect effects will be a key tool to build trust and to manage risk effectively.

The paper is structured as follows: in the next section we analyze the effect of petrochemical complexes on the risk perceived by the citizens and, by extension, on their health and well-being. Then we examine how citizens’ health risk perception may be influenced by trust (both in companies located in the petrochemical complex and in public institutions). This is followed by an explanation of the methodology and results. Finally, we discuss the main implications of the results and highlight the conclusions of the study.

## 2. Petrochemical Complexes, Risk and Health

Petrochemical complexes, frequently located in port areas close to cities, constitute an important focus of contamination through contact with chemical substances dumped into the water, air or soil [3,4,5,6,23,24,25,26]. The concentration of companies belonging to the petroleum and chemical industries in port areas comes as a result of the technical conditions of shipping transport, as well as the agglomeration economies arising from the location in a geographical area of firms with highly interrelated activities [27]. While these agglomeration economies generate benefits for the area, it is equally true that the geographical concentration of these industries results in some negative externalities that imply a number of significant risks, both in terms of possible accidents and diffuse contamination [28]. Occupational exposure studies have been frequently developed in order to analyze the risk perception and the health effects in workers of those facilities [29,30,31]. However, the potential exposure to a large set of chemicals may also be substantial for inhabitants living in nearby residential zones during the production and refining of crude oil and derivates, seriously affecting their health and well-being [26,28,32].

While, in general, the effects of environmental contamination on citizens’ health and well-being are extensively documented in the literature (see, for example, [33,34]), several studies have analyzed this issue in the specific context of petrochemical complexes, evaluating the effects of contaminants on certain physical diseases, risk perceived, psychosocial anxiety or fear [1,2,3,4,5,6,8,25,26,28,32]. As suggested by Luginaah *et al.* [5], catastrophic fears (e.g., explosions and fire), together with visual cues (e.g., flares, smoke stacks) create considerable anxiety, compounded by scientific uncertainty about the possible health impacts of refinery emission. Also, Cutchin *et al.* [8] indicated that petrochemical complexes are associated with increased risk, stress and self-reported illness. Moreover, it is important to point out that it is not necessary that individuals are conscious of the cause-effect relationship between their own health and the environmental problem. As argued by Mackerron and Mourato [35], given that awareness of environmental problems directly reduces life satisfaction, pollution in itself is an argument in risk perception, even independently of its actual health effect. In fact, individuals’ perceptions of environmental contamination are found to be positively associated with objective measures of contamination [36]. In summary, the relevant question is that the perceived risk of environmental threats associated to petrochemical complexes translates into specific psychological distress for exposed populations.

Moreover, the processes of expansion of the port industrial areas linked to the petrochemical sector exacerbate pollution problems and, consequently, future health problems in citizens. Petrochemical complexes usually comprise a wide range of companies that are likely to have a significant economic impact in their area of influence. In this type of activity, the interrelationship among companies within the complex is highly intense; to a greater or lesser degree, this concentration leads to agglomeration economies that generate economic benefits for the companies and, by extension, for the region in which they are located. In this kind of geographical location, the growth in economic activity is inextricably linked to increased levels of pollution and health problems, since petrochemical complexes tend to attract other similar companies. While at the same time they hinder diversification that would bring in companies from cleaner industries [37,38]. This question leads to consider on the benefits associated to the intensification of the growth of these industrial clusters (positive from an economic standpoint but negative from a public health approach). This phenomenon is clearly illustrated in Phillimore and Moffatt [38] who, referring to the Teesside petrochemical complex and the work of Banks [37], state: “as some of Banks’s interviews make apparent, chemical industry executives see one of the area’s attractions as stemming from the population’s experience of—and tolerance of—an industry that might face more of an uphill struggle in a setting with less historical familiarity. Thus, the difficult balancing act for those concerned with economic regeneration is to try to be both “green and clean” for diversification and simultaneously a place where continued petrochemical investment is welcome”. This circumstance is reproduced in the petrochemical complex that serves as empirical basis for our study.

## 3. Trust and Risk Perception

A considerable amount of research has been published on risk perception and a variety of theoretical perspectives have been adopted from sociological, psychological and cultural viewpoints, among others [39]. Risk perceptions are the subjective evaluations—the effective appraisal—of a hazard [8]. Consequently, risk perception is not just a matter of objective risk evaluation (typically based on the demonstrable probability of coming to harm, together with the severity of possible outcomes). The predominant paradigm in risk perception is that people are heavily dependent on industries and on the public institutions responsible for risk management in protecting citizens from possible harm [21]. A general assumption is that most people do not have sufficient knowledge of science and technology to be capable of judging risks, costs and benefits [40]. As a consequence people have to rely on others, which is why trust becomes important. Therefore, trust may be viewed as a mechanism to reduce the complexity faced by people [11].

Trust has become a popular research subject in the social sciences during the last two decades; it is thought to reduce social uncertainty and complexity [12]. Trust can be understood as “a psychological state comprising the intention to accept vulnerability based upon positive expectations of the intentions or behavior of another” [41]. It expresses the extent to which one expects the other to act in line with one’s own needs and interest. Trust has experienced growing visibility in risk-related research [17,42,43]. Most of these studies confirm the influence of trust on risk perception; some have even argued that the only concepts needed to describe risk perception are degree of trust and amount of outrage [44]. However, research into trust in this context is highly fragmented and difficult to classify systematically. The variety of approaches to define and measure trust may explain the conflicting results. It is clear that the relationship between trust and risk calls for additional examination, as evidenced by a number of current publication initiatives.

In the context of a pollutant industrial site as the one that concerns us, a petrochemical complex, individuals must rely on the intentions and competence of the industry itself—the companies located in the complex—and the public institutions that authorize and control its activity [14]. Trust in organizations and institutions responsible for risk management and communication may constitute an important factor influencing perception and acceptance of risks [45]. As we pointed out in the introductory section, in a review paper regarding literature on trust in the risk management field, Earle [18] shows that most of the articles analyzed that studied the consequences of trust included risk perception. Regarding the sign of the relationship, these studies show a negative effect when the referent of trust was responsible for managing the hazard, and positive when the referent was a critic of those responsible. More specifically, in the field of industrial risks, literature has shown that social trust has an important influence on risks perception of a nuclear waste repository [46,47], on risks associated with hazardous waste disposals [48], and on perceived risk of a chemical plant [43].

An important issue that has not been adequately resolved in the literature refers to operationalizing the social trust measure. For example, ter Huurne and Gutteling [21], when evaluating the responses of citizens towards industrial risks consider “institutional trust”, and include into this construct the trust of individuals in public institutions as well as the trust in the industry. Also Siegrist *et al.* [20], when operationalized “social trust”, they refer to the trust in both kinds of organizations, ultimately responsible for the proper management of risks. By contrast, Maeda and Miyahara [22] consider both kinds of trust, in the industry and in the government, as different variables and they estimate separate models to evaluate the determinants of each kind of trust. Similarly, the research of Peters *et al.* [49] concluded that the relative contribution of the determinants of trust (perceptions of openness and honesty; knowledge and expertise—competence; and perceptions of concern and care) differed for trust in industry and trust in government. Both kinds of trust, as it is shown in these studies and as we will discuss below, have a different basis and, consequently, we consider that trust in firms and trust in public institutions must be addressed separately in considering their impact on risk perception. Researches as Trumbo and McComas’ [15,16] simultaneously analyzed the incidence of both types of trust on the perceived risk, but they do not establish causality between the two kinds of trust. Therefore, studies to analyze the combined effect of these two types of trust, and to explore the relationship between them, are still lacking, and this is the basic purpose of the present research.

Drawing on the literature referred to as “causal chain model”, trust affects risk perception, and risk perception affects technology acceptance (see Earle [18] for a revision of different models of trust in risk management), although our work is limited to the first of these causal relationships. Both trust in the industry and trust in public institutions may, as discussed above, affect risk perception, although the basis is different. Companies are the first responsible for managing and communicating the risks inherent to the industrial activity which themselves developed. By contrast, the performance of public institutions in this field is associated with its regulatory activity as well as its function of authorizing and monitoring the industrial activity. Public institutions control that companies comply with environmental law, thus preserving the environmental quality of the surroundings. And beyond law enforcement, authorities develop actuations to reduce and control risks and to manage possible emergency situations. Hence, the foundation that underpins trust in the industry and trust in public institutions differs, which may have originated the differences in the contribution of their determinants in the studies of Peters *et al.* [49] and Maeda and Miyahara [22], justifying their separate treatment. However, with respect to their impact on the risk, and according to the “causal chain model”, one might expect a direct relationship, and with the same sign, between both variables—trust in companies and trust in public institutions—and risk perception, because these two kinds of organizations have responsibilities in risk management and communication. Consequently, and based on the above exposed, we propose the next hypotheses:

*H1:* *Citizens’ trust in companies located in the petrochemical complex influences directly, negatively and significantly their health risk perception.*

*H2:* *Citizens’ trust in public institutions influences directly, negatively and significantly their health risk perception.*

However, both kinds of trust are not independent, as shown, for example, in the works of Trumbo and McComas [15,16], where it is found that there is a positive correlation between them. However, neither in these works nor in others, as far as we have seen, the causal relationship between these two variables is analyzed, a question which we will try to address below.

The level of trust between a firm and the members of the community may be a function of the information asymmetry between them regarding the firm’s environmental practices and its environmental performance [50]. The quality of environmental management is not readily observable, and consequently this is an area characterised by strong information asymmetries. In this context, trust in industry can come largely conditioned by trust in public institutions in exercising its role of monitoring environmental practices of companies and enforcing the law. According to institutional theory, organizations recognize the importance of achieving social legitimacy for their long-term survival [51,52], and law enforcement is a source of corporate legitimacy [53,54]. Ultimately, legitimacy represents a vital resource for the sustained survival of companies in competitive environments, and organizational legitimacy is usually based on compliance with social rules (*i.e.*, laws and formal regulations).

The public authorities have implemented a number of regulations in recent decades in response to EU Directives 96/82/CE-Seveso II (acute risk) and 96/61/EC (chronic risk) and amendments. The Council Directive 96/61/EC concerning integrated pollution prevention and control (the IPPC directive) has as one of the principal objectives the use of the Best Available Practices (BATs), to protect the environment as a whole [55]. The companies located in the petrochemical complex are regulated by these directives and requires the pertinent authorization in order to start and maintain their activity. This authorization sets environmental conditions that are required for the operation of firms’ facilities and also it specifies the emission limit values of pollutants, which will be based on the BATs. Public institutions are responsible for the approval of the installation of companies, which has repercussions in the enlargement processes of the petrochemical complex, and also to monitor compliance with environmental obligations by companies that are already established, executing appropriate sanctions for non-compliance. To the extent that public institutions exercise their role properly, it is increased the guarantee that companies will be acting correctly regarding to their environmental practices, at least in regard to law enforcement and the use of the BATs. Consequently, trust in public institutions, in their role as enforcing the law and discouraging misconduct by companies regarding their environmental behavior while guaranteeing public health, can lead to legitimate companies, considering that they are doing the right things and, consequently, this may increase trust in the companies themselves. Based on this argument, our study analyzes the relationship between citizens’ trust in companies and public institutions and their risk perception, and we propose the next hypothesis:

*H3:* *Citizens’ trust in public institutions influences directly, positively and significantly their trust in companies located in the petrochemical complex.*

**Figure 1:**
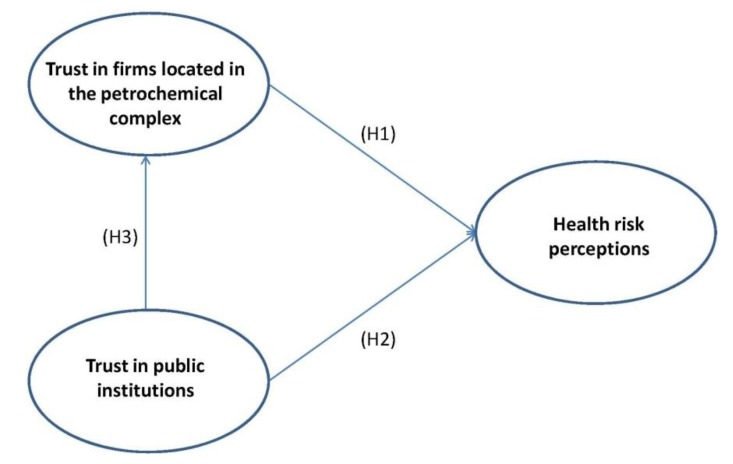
Causal model hypothesized for trust-risk relationship.

In summary, the research model is showed in Figure 1. The three hypotheses build up the model, linking trust in firms located in the petrochemical complex, trust in public institutions and citizens’ health risk perception.

## 4. Methodology

### 4.1. Scope of the Study

The area of our analysis is the petrochemical complex located in the port of Castellon (Spain), that is known as El Serrallo industrial estate, currently covering a total area of 3,635,400 m^2^, in the town of Castellón (UTM coordinates: X-755 803, 688 Y-4426958, 037). Today it accommodates nine companies, most of them belonging to the petrochemical sector. These companies are: (1) an oil refinery belonging to a global energy company whose principal activities are exploration and production of oil and gas, refining and marketing of both raw materials and alternative energy; (2) a chemical company producing caprolactam, fertilizers, liquid manures and ammonium sulphate; (3) a company retailing liquefied petroleum gas; (4) a company serving the oil refinery engaged in transporting and storing petroleum products; (5) a electricity generating company that operates a combined cycle plant; (6) a hazardous waste treatment company that emerged from the agreement between an industrial waste management company and the refinery; (7) a public terminal for the unloading, storage and dispatch of bulk goods; (8) a plant for grinding clinker and the production and operation of cement; (9) a vegetable oil-based biodiesel production plant. The last three companies mentioned were installed in the estate from the last enlargement made, opened in 2009, which had 1,000 million Euros of investment (public and private funds). This expansion represented expanding the installation more than 2,000,000 m^2^. So next to the businesses already established, the purpose is to install new industrial activities to completely fill the new port lands resulting from enlargement.

### 4.2. Data Collection

Data about risk perception and trust were gathered by a questionnaire administered to a representative sample of citizens located in the residential area surrounding the petrochemical industry situated in the port of Castellón. The fieldwork was carried out in March and April, 2011, in order to concentrate responses in a short space of time. Face-to-face street interviews were conducted with residents to complete the questionnaire, which consisted of closed questions with items measured on a five-point Likert scale, where 1 represented the lowest agreement with the statement, and 5 the highest. A total of 992 valid responses were obtained using simple random sampling. Of these responses, 542 (54.6%) were from women and 450 (45.4%) from men. The majority of the respondents reported an age of between 26 and 45 (48.4%); young surveyed, from 18 to 25 years, were 128 (12.9%); respondents from 46 to 65 years were 286 (28.8%), while the lowest age range represented was the over 65 bracket, 98 interviewees (9.9%). Lastly, a total of 521 (52.5%) respondents reported an intermediate level of education, 307 (30.9%) a lower level and 164 (16.6%) a higher level of formal education. According to official Spanish census data [56], the total population of the area surveyed is 42,086, which for a 95% confidence level, represents a sample error of ±3.10% (*p* = *q* = 0.5) for the whole sample.

The scale items used to measure the variables of the study were adapted from previous studies: risk perception [15], trust in industry and trust in public institutions [21]. Regarding trust, we use basically the items of the work of ter Huurne and Gutteling [21], which captured the different components of trust including openness, credibility, expertise and concern, but we extended the measurement scale for trust in public institutions, using as reference the work of Poortinga and Pidgeon [12], to assess the integrity of these institutions. Prior to drafting the final questionnaire with all the items, we carried out a series of in-depth interviews in order to design the final questionnaire that we would use to obtain residents’ assessments. These interviews were held with appropriate members of the community who represented the various stakeholder groups in the area (two employees from the chemical industry, a representative of the fishing sector and several members of a local neighborhood association). An initial version of the questionnaire was drafted following these interviews. Regarding the construct of risk perception, for which we take as reference the work of Trumbo and McComas [15], we removed one of the items, which referred to the risk control by the subject, since according to the personal interviews we considered it was not relevant in the context analyzed. Once the results from these interviews were processed, the final questionnaire was pre-tested, which provided quantitative data in the form of assessments with the proposed scales, and qualitative data, from the analysis of opinions on more formal issues of the questionnaire. The final version of the items included in the questionnaire is provided in the Appendix.

### 4.3. Statistical Procedure

The model was empirically validated using Structural Equation Modeling (SEM). This method takes into account the existence of measurement error and allows all the relationships proposed in the theoretical model to be estimated simultaneously, thus giving a complete representation of the model. SEM is therefore a suitable methodology to test, in a single model, the relationships between the trust (both in companies and publics institutions) and citizens’ risk perception.

We used the EQS 6.1 [57] statistical software package, with the maximum likelihood estimation method; to protect our results from possible deviation from the assumption of normality, all the Chi-square values (and standard errors) that appear correspond to the Satorra and Bentler [58] goodness of fit statistics. To evaluate the goodness of fit of the models, given the possible non-normal distribution of the data analyzed, we used the Satorra-Bentler scaled Chi-square statistic [57,58], following previous studies that have used this modification of the statistic [59].

## 5. Results of the Empirical Study

### 5.1. Validation of the Scales

To ensure the dimensionality, reliability, and the convergent and discriminant validity of all the scales used in the study, we performed the series of analyses described below that allowed the scales to be refined by eliminating non-significant items, using Confirmatory Factor Analysis with the Structural Equations Modelling (SEM) technique. After that, we established the causal structure, enabling the causal hypotheses to be tested.

#### 5.1.1. Dimensionality

The dimensionality of the scales was verified using an overall Confirmatory Factor Analysis for all the items of the model, taking into account the variables of each one of the items. The goodness of fit of the model (see Table 1) was above the recommended values in all cases (χ² (101) = 412.3404; CFI = 0.972; RMSEA = 0.056; BBNFI = 0.964; BBNNFI = 0.967), verifying that each item only forms part of its corresponding variable. In addition, only one item had a factor loading below 0.50, but as it did not lead to problems in the reliability of the construct, we decided to keep it as part of the original scale. Additional analyses conducted deleting this item showed very similar results in terms of reliability than those presented below, showing that it was better not to definitely delete the item avoiding problems on the content validity of the model.

**Table 1:** Dimensionality, reliability and validity of the scales.

ITEMS	Loading	T	Mean	Standard deviation
Trust in companies located in the petrochemical complex				
AVE = 0.76; CR = 0.94; Cronbach’s Alpha = 0.937				
P1.1	0.87	Fixed	2.27	1.22
P1.2	0.86	39.99	2.15	1.32
P1.3	0.93	49.96	2.22	1.22
P1.4	0.78	32.21	2.84	1.38
P1.5	0.90	37.18	2.06	1.16
Trust in public institutions				
AVE = 0.66; CR = 0.93; Cronbach’s Alpha = 0.923				
P2.1	0.88	Fixed	2.08	1.20
P2.2	0.89	47.58	1.96	1.178
P2.3	0.92	47.96	2.14	1.21
P2.4	0.74	26.54	1.76	1.06
P2.5	0.38	12.01	2.24	1.19
P2.6	0.90	38.31	2.05	1.16
P2.7	0.85	36.87	2.11	1.22
Health risk perception				
AVE = 0.68; CR = 0.89; Cronbach’s Alpha = 0.889				
P3.1	0.88	Fixed	3.51	1.47
P3.2	0.83	31.54	3.04	1.52
P3.3	0.89	43.95	3.57	1.46
P3.4	0.67	25.78	3.09	1.61
Fit of the model:				
Chi-square (S-B) = 412.34; d.f. = 101; Chi/d.f. = 4.082				
CFI = 0.972; RMSEA = 0.056; BBNFI = 0.964; BBNNFI = 0.967				

#### 5.1.2. Reliability

Cronbach’s alpha [60] and composite reliability [61] were used to confirm the reliability of the scales. Table 1 presents the values of the two indicators for each scale. Values are above the recommended minimum of 0.7 in all cases [62], with the scales for trust in companies and trust in public institutions presenting notable values equal to and above 0.9, respectively.

#### 5.1.3. Convergent and Discriminant Validity

Convergent validity was evaluated using the Bentler-Bonett normed fit index (BBNFI) [63]. The BBNFI is the index of the difference between the Chi-square of the model minus the Chi-square of the null model (the independent model in which all correlations are equal to zero), divided by the Chi-square for the null model. A BBNFI over 0.90 indicates strong convergent validity [64]. In addition to this analysis, the variance captured by the construct indicators in relation to the average variance explained (AVE) of the variables of the model is higher than the recommended minimum of 0.5 [61]. Considering the cut-off value for the BBNFI and the recommended value for the AVE, displayed in Table 1, the model analysed presents a high level of convergent validity.

Finally, the discriminant validity of the model was confirmed by verifying that the square root of the AVE of all the variables of the model was greater than its correlation with the other variables, as shown in Table 2. In light of the above, the convergent and discriminant validity of the variables included in the model are demonstrated.

**Table 2:** Discriminant validity of the model variables.

Discriminant validity	Tr_C	Tr_PI	R_P
Trust in companies	0.87		
Trust in public institutions	0.66	0.81	
Health risk perception	−0.48	−0.29	0.82

#### 5.1.4. Descriptive Statistics

Prior to analyzing the structural model, note that Table 1 shows the averages for each item and the standard deviation of the responses to each item. In Table 2 the correlations between each of the variables are reported, and in all cases these are different from zero, and positive or negative in accordance with the wording of the items for each of the variables (see the appendix).

### 5.2. Results of the Structural Model

After analyzing the scales, the empirical testing of the model concludes with the analysis of the relationships hypothesized that, together, make up the model tested in this study. Last row of Table 1 reports the goodness of fit indexes for the structural relationships model proposed. Various statistics were used to assess the goodness of fit of the model [65]; all the values of these statistics were adequate, thus verifying the model’s suitability for the sample. In summary, this demonstrates that the structure of the relationships among variables proposed to explain citizens’ health risk perception in the vicinity of an industrial estate is valid for the dataset obtained.

**Figure 2:**
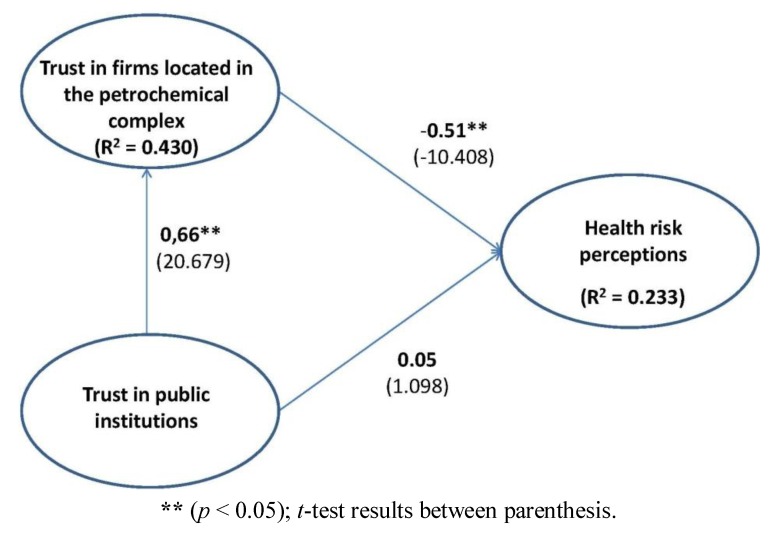
Results for the structural equation model.

Figure 2 shows the estimated parameters and the t-tests corresponding to the weightings of each of the relationships considered in the model explaining residents’ health risk perception. The results of the estimation show a significant causal relationship between the trust in companies and the citizens’ health risk perception, and between the trust in public institutions and the trust in companies, which allows us to accept H_1_ and H_3_. However, the direct effect between the trust in public institutions and the risk perception is not significant (H_2_ is not corroborated). Furthermore, the results of the coefficient of determination for health risk perception (R^2^ = 0.233) reveal that the antecedent variables considered explain 23.3% of the variance of perceived risk. The variance of the other dependent variable—trust in companies of the petrochemical complex—is explained by the trust in institutions with a value of 43.0%. Finally, our analysis asked if significant indirect effects would be present between trust in public institutions and risk perception in a path model, with the trust in companies as intervening variable between trust in public institutions and risk perception. EQS also provides a convenient facility for effects decomposition. Table 3 presents these results, which show that indirect effects are present for the trust measure of public institutions. For calculation of the significance of decomposed effects see MacKinnon [66].

**Table 3:** Effects decomposition for prediction of risk perception by trust in public institutions.

	Trust in public institutions
Total effects	−0.29 **
Direct effects	0.05
Indirect effect via trust in companies	−0.34 **
Indirect effects as percentage of total	85.29

** (*p* < 0.05).

## 6. Discussion and Conclusions

Petrochemical industrial complexes (oil refineries are commonly located at industrial poles together with other pollutant chemical firms) constitute an important focus of pollution. Residing in the vicinity of these sites, which contribute to the environmental deterioration of their geographical surroundings, contributes to undermine quality of life. The potential exposure to contaminants contributes to create uncertainty, psychosocial anxiety and distress which have a wide range of effects on health and well-being. Specifically, citizens who are confronted with this kind of industrial complex try to form fair judgments about the risks they are exposed, but the information is often limited or they have not the capabilities to understand it. Under such circumstances, citizens’ perceptions and feelings about the agents responsible for managing these risks—companies located in the petrochemical complex and public institutions—play a dominant role. Citizens’ trust in organizations responsible for risk management and communication constitutes an important factor influencing perception and acceptance of risks and its importance has been recognized in the literature in the field of risk management.

The main goal of this paper was to evaluate the combined effect that trust in firms located in the petrochemical complex and trust in public institutions has on citizens’ health risk perception, considering simultaneously the relationship between both dimensions of social trust. Our research produced two major findings. When both kinds of trust were treated jointly in the same model, differences were encountered in the trust-health risk perception relationship depending of the type of trust considered. Thus, trust in companies located in the petrochemical complex, as agents responsible in the first place to manage the risk of industrial activities developed, has a negative impact on citizens’ health risk perception. However, trust in public institutions, which are the regulatory authorities and also take the responsibility on the supervision and control of the companies regarding to their environmental performance, has not a direct and significant relationship with health risk perception. Finally, the most important result of this research is that trust in firms is a significant mediating variable in the indirect effects that trust in public institutions induces in health risk perception. Consequently, trust in public institutions, when both kinds of trust are considered jointly, has not retained a direct effect on citizens’ health risk perception but exert an indirect causal link—through trust in firms. These findings contribute in a relevant way to the literature on social trust and health risk perception.

These results have some relevant implications for the firms located in the complex and also for the public institutions responsible for controlling their activities. Our results may be useful for managers of the firms of this petrochemical complex in relation to their behavior and their communication policy. They ought to be concerned about the level of trust showed by residents, with responses in all five items representing trust in companies with average values below the middle of the scale. Regularly negative consequences generated by a petrochemical complex, such as air pollution, noise, garbage, and/or sporadic episodes of oil spills related to its activity, contribute highly to create a bad image in residents’ minds. Given this fact, and taking in consideration the negative relationship between trust in companies and citizens’ health risk perception, the problems associated to the residents’ stress and psychological anxiety will be seen certainly accentuated. Consequently, it is necessary a drastic change in the culture of the firms integrate this petrochemical complex, based on how to manage and communicate risk and encourage the trust of residents.

Also of concern are the findings for public institutions. It is also prominent the lack of trust in public institutions as regulatory authorities and also in its role of monitoring companies’ environmental behavior and ensuring the health and the well-being of citizens. Regarding to this, and as shown in the results of our research, public authorities should be aware that the trust citizens place in them has a significant influence on trust aroused by companies in their activity. If public institutions properly exercise its regulatory function and induce firms to correct environmental behavior (for example, establishing penalties when their actions are not appropriate), the citizens’ trust in companies will also be strengthened. Again, as in the case of trust in firms, the average values of the responses were below the middle of the scale for all items. Distrust can be in great part due to the insensitivity of authorities in addressing residents’ environmental concerns, a fact that again reinforces the need to establish appropriate relationships with the community. In addition, from a sociological perspective, the growing public distrust in government and business in recent years of severe financial crisis found in various surveys in Spain should also be noted. In any case, both companies and public institutions should focus their efforts on shoring up public trust, reducing risk perception and ultimately improving the health and well-being of residents. Generating and maintaining trust often constitutes a primary goal of the industrial communication policy.

The issue of cumulative effects of pollution in the whole complex and its impact on the perceived risk plays an important role in combining the interests of industrial and health policies (for example, in the growth of the petrochemical complex). Thus, the results should prompt reflection on the part of the port authorities and the other relevant institutions responsible for territorial planning regarding this enlargement. Such enlargements are often necessary to maintain the levels of competitiveness of the cluster and of the companies located in them. Indeed, new companies are currently being considered to join the petrochemical complex analyzed in this study. One of those firms is a large-scale fertilizer plant that has aroused debate among residents over its suitability for inclusion in the industrial estate. The very nature of an industrial complex is such that it acts as a magnet for new companies with links to the petrochemical sector, and it is extremely complicated to develop initiatives that encourage diversification of the industrial activity, which hinders the flow of investment into other sector types. Public institutions should be aware that these enlargement processes will contribute to increase perceived risk and to greater psychological distress of citizens as consequence of cumulative effects of pollution. It is necessary in a context like the present one that companies should redouble their efforts to what might be called “shared responsibility”, intensifying its activities in the environmental field to ensure the health and the welfare of the residents. Health risks resulting from the cumulative effects of different environmental sources constitute an essential component of risk management decisions aimed at protecting residents [67,68,69]. Residents exposed to potential chemical hazards are interested in what policies responsible agencies in risk management enact to reduce the risk and ensure a safe environment.

Finally, although the results of our research are encouraging, they are tempered by the limitations of the research. As the first limitation of our study, we must point out that the results obtained are contingent on the context analysed. Consequently, we note that the results may not be generalizable, since they refer to a very specific location in a single petrochemical complex. In further studies, an analysis of other realities would also be of interest to compare the results obtained. Finally, we stress that this is a cross-sectional analysis, which opens the way for future research to obtain the same data for different years, and to address a longitudinal analysis which may provide significant conclusions about the evolution over time of social trust and risk perception.

## Appendix

**Table:**

Initial scales used to measure the variables of the model	
Trust in companies located in the petrochemical complex	
P1.1	These companies protect local residents from possible harm deriving from their activities.
P1.2	I believe these companies when they say that they do as much as possible to minimise the risks to residents.
P1.3	These companies are concerned about the safety and health of citizens.
P1.4	These companies know how to handle the risks deriving from their activities.
P1.5	These companies listen to and are sensitive to the environmental worries of residents.
Trust in public institutions	
P2.1	Public authorities protect residents from any damages arising from the activities of companies in the industrial estate.
P2.2	I believe public authorities when they say that they do everything to minimise risks to residents.
P2.3	Public authorities are concerned about the safety and health of citizens.
P2.4	Public authorities openly report on environmental risks of the industrial estate to citizens.
P2.5	Public authorities are heavily influenced by the companies in the industrial estate when evaluating environmental risks (*reversed*).
P2.6	Public authorities listen and are responsive to environmental concerns of residents.
P2.7	Public authorities act in favour of the public interest on issues concerning environmental contamination.
Health risk perception	
P3.1	I believe my health is exposed to risks by living in this area.
P3.2	I frequently worry about the risks related to living in this area.
P3.3	I am concerned that living in this area poses risks that will extend to future generations.
P3.4	The risks to health associated with living in this area have increased in recent years.
